# Exercise and *spirulina *control non-alcoholic hepatic steatosis and lipid profile in diabetic Wistar rats

**DOI:** 10.1186/1476-511X-10-77

**Published:** 2011-05-15

**Authors:** Leandro P Moura, Guilherme M Puga, Wladimir R Beck, Inaian P Teixeira, Ana Carolina Ghezzi, Gláucio A Silva, Maria Alice R Mello

**Affiliations:** 1 São Paulo State University "Júlio de Mesquita Filho". Department of Physical Education. Institute of Biosciences. Laboratory of nutrition, metabolism and exercise. Av 24A, 1.515, Bela Vista - 13.506-900 - Rio Claro, SP, Brazil

## Abstract

**Background:**

Diabetes mellitus is associated with metabolic dysfunctions, including alterations in circulating lipid levels and fat tissue accumulation, which causes, among other pathologies, non-alcoholic fatty liver disease (NAFLD).

**Aim of the study:**

The objective of this study was to analyse the effects of physical exercise and *spirulina *intake on the control of NAFLD in diabetic Wistar rats.

**Methods:**

Diabetes was induced in the animals through intravenous administration of alloxan. The rats were divided into four groups: Diabetic Control (DC) - diabetic rats fed with a control diet and no physical exercise; Diabetic *Spirulina *(DS) - diabetic rats fed with a diet that included *spirulina*; Diabetic *Spirulina *and Exercise (DSE) - diabetic rats fed with a diet that included *Spirulina *and that exercised; and Diabetic Exercise (DE) - diabetic rats fed with a control diet and that exercised.

**Results:**

The groups DS, DSE, and DE presented lower plasma concentrations of LDL cholesterol than DC, as well as lower levels of total liver lipids in groups DS, DSE, and DE in comparison to DC.

**Conclusion:**

Thus, *spirulina *appears to be effective in reducing total circulating levels of LDL-cholesterol and hepatic lipids, alone or in conjunction with physical exercise in diabetic rats.

## Introduction

Type 1 diabetes (DM-1), is an autoimmune illness that primarily affects young people. Because patients with type 1 diabetes do not produce sufficient quantities of insulin, they are dependent on exogenous insulin to maintain blood glucose at normal levels. In turn, type 2 diabetes (DM-2) occurs more slowly and later in life (after forty years of age) than type 1.

The complications associated with diabetes are severe. The illness is one of the main causes of blindness, kidney disease, macrovascular disease and atherosclerosis, liver disease and a variety of debilitating neuropathies that diminish the quality of life and life expectancy of the patients [1]. The imbalance of the circulating lipid profile is a consequence of diabetes mellitus [2]. According to Carew *et al*. [3], the imbalance in the concentration of serum lipoproteins can promote the movement of cholesterol from peripheral tissues to the liver for catabolic excretion. In cases where this transference to the liver is high and the catabolic level is low, fat can accumulate in this organ, causing non-alcoholic fatty liver disease (NAFLD). Furthermore, chronically high concentrations of serum lipids associated with a low level of catabolism can create the same condition. This illness is characterised by the presence of large depots of fat in the liver and the absence of inflammatory processes and is known as hepatic steatosis [4].

In addition to dietary control, physical exercise has been heavily used as a non-pharmaceutical treatment for the control and reduction in abnormal levels of circulating lipids and glucose in individuals with metabolic dysfunctions such as diabetes [5,6].

From this perspective, functional foods have been used for the control of these metabolic dysfunctions. Some authors have demonstrated that *spirulina *intake can regulate cholesterol, increase the antioxidant capacity, and improve insulin resistance and the uptake of glucose [7-9].

*Spirulina *is a helical blue-green alga, which contains 65 to 70% protein [10,11]. Although it has a slightly reduced digestibility [12], it appears to be a good food source of protein. In addition, some studies show that *spirulina *has beneficial effects on the treatment of malnutrition [13,14] and on other pathologies such as obesity [13,15], hypercholesterolaemia [13,11], arterial hypertension [13,16,17] and diabetes mellitus [13].

In agreement with Hosoyamada *et al*. [9], the water-soluble fraction of *spirulina *is effective in diminishing serum glucose levels in fasting rats [12] due to phycocyanin, which is capable of reducing concentrations of circulating glucose [13]. According to studies by Layam and Reddy [18], when this alga is administered at a concentration of 15 mg/kg body weight, it can raise serum insulin levels in DM-1 rats.

Given the difficulties encountered in studies that involve hepatic steatosis in human subjects, several studies have been performed using animal subjects, which permits researchers strict control of nutrition and environment and also the analysis of specific tissues, such as hepatic and myocardial tissues. Thus, the objective of the present study was to verify the effects *spirulina *intake and of swimming training on the lipid profile, in hepatic steatosis and on the accumulation of lipids in myocardial and skeletal muscle of diabetic Wistar rats.

## Materials and methods

### Animals and Treatment

The study used young Wistar rats (60 days old), which came from the Central Vivarium (Biotério Central) of UNESPA - at the Botucatu Campus and were maintained in the vivarium of the biodynamic laboratory of the Department of Physical Education, Biosciences Institute of UNESP - Rio Claro Campus. The animals were housed in polyethylene cages (five animals per cage) and kept at a controlled room temperature of 23 ± 1°C, with a photoperiod of 12 hours of light/12 hours of dark with food and water *ad libitum*. The experiment was conducted in accordance with current Brazilian legislation and standards of the Brazilian College of Animal Experimentation (Colégio Brasileiro de Experimentação Animal-COBEA). All standards were adhered to rigorously. The use of animals in this study was approved by the ethics committee at the Biosciences Institute at UNESP - Rio Claro (protocol 5111 of 08/20/09).

### Induction of Diabetes

Experimental diabetes was induced by administration of alloxan monohydrate (Sigma, 32 mg.kg^-1 ^of body weight) dissolved in citrate 0.01 M, pH 4.5, injected into the penile vein. After this procedure, the animals were returned to their cages and received, in the first 24 hours post-alloxan, a solution of water and glucose (15%), in addition to feed [19]. Two weeks after administration of the drug, the blood glucose level of the animals was tested for evidence of a diabetic state. The animals that had a blood glucose level equal to or greater than 190 mg.dL^-1 ^were included in the study.

### Experiment Design

After the induction of type 1 diabetes, the animals were randomly distributed into four groups (10 per group): Diabetic Control (DC) - composed of alloxan diabetic rats that were given a control diet and no exercise; Diabetic *Spirulina *(DS) - composed of alloxan diabetic rats fed with a diet that included *spirulina *and no exercise; Diabetic *Spirulina *and Exercise (DSE) - composed of alloxan diabetic rats fed with a diet that included *spirulina *and that exercised; Diabetic Exercise (DE) - composed of alloxan diabetic rats fed with a control diet and that exercised.

### Diets

Following the recommendations of the American Institute of Nutrition AIN-M93 [20], isocaloric and semi-purified diets were utilised; the control diet for the animals in the control (DC) and exercise (DE) groups contained 17% casein protein, and the rats in the *spirulina *groups (DS and DSE) were fed with a diet containing 17% *spirulina *protein. The control diet was composed of the following (%): corn starch = 39.7, dextrin = 13.2, sucrose = 10, soy oil = 7, microcellulose = 5, a mixture of six minerals = 3.5, and a mixture of vitamins = 1. A detailed description of the mineral and vitamin mixtures can be found in Reeves *et al*. [20]. The *spirulina *diet contained the same quantities of carbohydrates and lipids as the control diet. There were adjustments made only in the contents of the six minerals, the vitamins, and the fibres, taking into account the levels of these elements already in the *spirulina*, so that the recommendations of AIN-M93 for these nutrients would be maintained [21].

### Adaptation to the liquid medium and exercise protocol

In the first week, the animals were put into contact with shallow water for adaptation to the liquid medium. From the second week, they had swimming exercise with increasing amounts of time. The training sessions were held in collective tanks with a constant water temperature of 31 ± 1°C, for 1 hour/day, 5 days/week, for a total period of 44 days. During the exercise, a backpack was attached to the thorax of the animal with a load equivalent to 3.5% of body weight. This protocol was selected to represent aerobic exercise below the lactate threshold for diabetic rats [22].

### Assessments of animals prior to their sacrifice

During the entire experimental period, all the animals had their body mass, water and food intake registered. The results were analysed calculating the area under the curves in the variations of body mass and food intake over time using the trapezoidal rule [23].

### Assessments of animals after their sacrifice

At the end of the experimental period, 48 hours after the final "in vivo" evaluations, at feed and rest conditions, all the rats were anaesthetised in a CO_2 _chamber until they were sedated. They were then exsanguinated, and their blood was collected. The blood was centrifuged at 3000 rpm for 15 minutes, and analyses of glucose, total cholesterol, triglycerides, HDL cholesterol, LDL cholesterol, total protein, and albumin were performed using samples of supernatant serum, through colourimetric methods with commercial colourimetry test kits (Laborlab^®^, Guarulhos - SP/Brazil). Free fatty acid (FFA) were also analysed according to the Regow method [24], and insulin was measured by radioimmunoassay [25].

Samples of the left ventricle, the liver, and the gastrocnemius muscle were taken to determine the concentrations of triglycerides and total lipids. The samples were placed into tubes containing 0.1% Triton X-100. They were then homogenised with a Polytron^® ^for 20 seconds at a maximum velocity. After this procedure, the samples were centrifuged at 4.000 rpm for 10 minutes. The supernatant was extracted to determine the triglycerides and total lipid using spectrophotometry with a commercial kit (Laborlab^®^, Guarulhos - SP/Brazil).

### Statistical Analysis

The results are expressed as means and standard deviations. After checking the data for normalcy using the Shapiro-Wilk test, the values of the lipid profile, plasma proteins, adipose tissue proteins, areas under the curve of variations in body mass and the intake of food and water during the experiment were compared between the groups using analysis of variance - one way ANOVA with Tukey's post-hoc test. The adopted significance level was p < 0.05.

## Results

**Figure 1:**
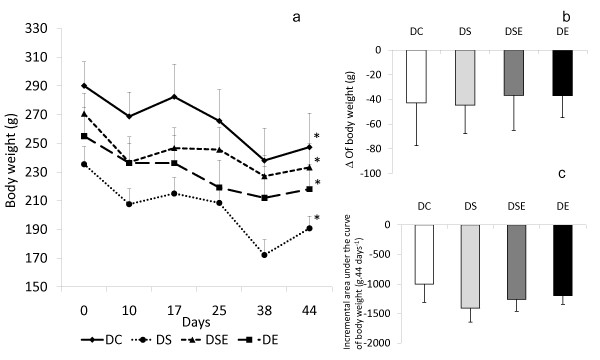
**Mean and standard error of body mass** - a; the difference between body mass pre- and post-intervention (Δ = variation of mass in relation to initial mass over time) - b; area under the curve of body mass over time of the body mass - c. DC - control group; DS - group with *spirulina *intake and without exercise; DSE - group with *spirulina *intake and with exercise; and DE - group without intake of *spirulina *and with exercise (10 animals/group).

The results of body mass in each group are shown in Figure 1. All groups had significantly reduced body mass by the end of the 44 days of training in relation to pre-intervention values as indicated by the area under the curve of body mass over time (Figure 1-a). Furthermore, the statistical analysis did not show a significant difference between the groups regarding the change (Δ) in body mass (final value of body mass - initial value of body mass) during the experiment, as shown in Figure 1-b. There was also no difference between the groups when the values of the area under the curve of body mass were compared, as demonstrated in Figure 1-c.

**Figure 2:**
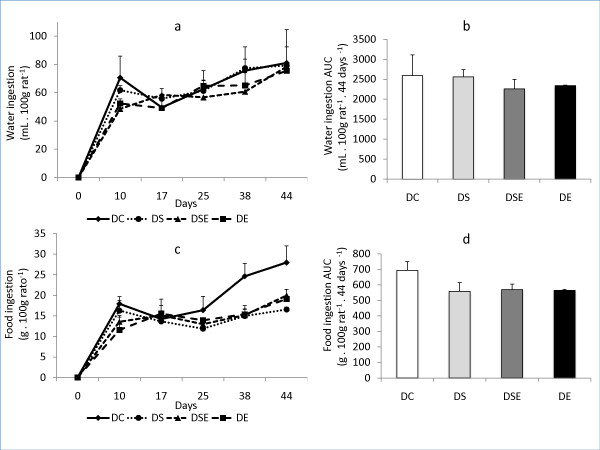
**Mean and standard error of water consumption (a) and area under the curve (b) of all groups**. Median values of food consumption (c) and the area under the curve (d) of all groups. DC - control group; DS - group with *spirulina *intake and without exercise; DSE - group with *spirulina *intake and with exercise; and DE - group without intake of *spirulina *and with exercise (10 animals/group).

The results of water intake in each group are illustrated in Figure 2-a. There was no statistical difference between the areas under the curve of water intake over time (Figure 2-b) when comparing all groups. The statistical analysis also did not show a significant difference between areas under the curve of food intake when comparing all groups (Figures 2-c and 2-d).

The results of serum concentrations of glucose, insulin, total, HDL and LDL cholesterol, triglycerides, FFA, total proteins, and plasma albumin of all groups are presented in Table 1. For the concentrations of serum insulin, the DS group had a significantly lower value when compared to the DC, DE, and DSE groups. The statistical analysis did not show differences in concentrations of glucose, total cholesterol, triglycerides, FFA, or total proteins in the groups studied.

**Table 1:** Serum analysis at the end of the experiment.

	DC	DS	DSE	DE
Total Cholesterol (mg.dL^-1^)	108.8 (9.8)	105.5 (4.9)	94.3 (6.2)	91.2 (4.0)
HDL - Cholesterol (mg.dL^-1^)	19.8 (0.8)	17.7 (0.7)	15.7 (1.0)*	18.4 (0.7)
LDL - Cholesterol (mg.dL^-1^)	64.0 (7.3)	42.2 (5.7)*	37.4 (3.9)*	52.2 (3.8)
Triglycerides (mg.dL^-1^)	105.6 (9.9)	237.2 (39.9)	237.8 (46.4)	112.7 (22.2)
Free Fatty Acids (mg.dL^-1^)	0.66 (0.05)	0.66 (0.06)	0.62 (0.04)	0.67 (0.04)
Albumin (mg%)	2.61 (0.06)	2.42 (0.02)	2.28 (0.14)*	2.56 (0.06)
Total Proteins (mg%)	7.66 (0.36)	7.19 (0.11)	7.34 (0.32)	7.47 (0.19)
Glucose (mg.dL^-1^)	303.20 (70.69)	289.25 (106.65)	248.88 (84.92)	275.40 (90.90)
Insulin (U.mL^-1^)	1.64 (0.21)	0.43 (0.21)*	12.69 (3.24)	9.73 ± 3.68

Results expressed with means and (standard errors) of 10 animals/group. DC: Diabetic Control, DS: Diabetic *Spirulina*, DSE: Diabetic *Spirulina *Exercise and DE: Diabetic Exercise. Statistical Difference (ANOVA p < 0.05), *= different from DC.

The concentrations of LDL cholesterol were 34 and 42% less in the DS and DSE groups, respectively, in relation to the DC group. The HDL cholesterol levels were 20% lower in the DSE group in relation to the DC group. Furthermore, the concentration of albumin was 17% lower in the DSE group in relation to the DC group.

The results of the tissue concentrations of lipids are presented for all groups in Table 2. The statistical analysis did not show a significant difference in triglyceride concentrations in muscular tissue or in the left ventricle myocardial groups but did show that the concentration of hepatic triglycerides was 43% less in the DS group in relation to the DC group. In turn, the concentrations of total hepatic lipids were 46, 44 and 43% lower in relation to the DC group for the DS, DSE and DE groups, respectively.

**Table 2:** Tissue lipids analyses at the end of the experiment.

	DC	DS	DSE	DE
***Liver***				
Total Lipids (mg%)	07.61 (1.52)	04.15 (0.17)*	04.24 (0.29)*	04.36 (0.49)*
Triglycerides (mg%)	18.52 (3.87)	10.59 (1.05)*	11.11 (1.32)	12.12 (1.94)
***Gastrocnemius Muscle***				
Triglycerides (mg%)	12.04 (1.60)	08.82 (0.95)	08.38 (0.84)	09.17 (0.98)
***Left Ventricle***				
Triglycerides (mg%)	5.95 (0.59)	07.07 (0.67)	06.27 (0.59)	6.43 (0.70)

Results expressed with means and (standard errors) of 10 animals/group. DC: Diabetic Control, DS: Diabetic *Spirulina*, DSE: Diabetic *Spirulina *Exercise and DE: Diabetic Exercise. Statistical Difference (ANOVA p < 0.05), *= different from DC.

## Discussion

This study aimed at assessing the effects of *spirulina *intake and physical exercise on circulating and tissue lipid levels (hepatic, myocardial, and skeletal muscle) in diabetic Wistar rats. The principal finding of our study was that *spirulina *in the diet of these rats resulted in an attenuation of fatty deposits in the liver as well as lower plasma levels of LDL cholesterol in comparison to the diabetic control group that did not receive treatment.

In fact, the literature has shown conflicting results in studies including a diet with *spirulina *and exercise. One of the first studies with rats consuming *spirulina *showed a reduction in total cholesterol levels when examining the lipid profile [26]. Since then, various studies have been conducted using animal [17,27,28] and human models [29,30].

In relation to the intake of water and food, the statistical analysis showed that there was no significant difference between groups (Figure 2). Furthermore, total protein concentrations and serum albumin that can be used as measures of the nutritional and hydration states of animals showed values quite similar to those already reported by other authors that also study active and sedentary diabetic Wistar rats [31].

Using an experimental model, Kato *et al*. [27] submitted rats to a diet rich in cholesterol with and without *spirulina *supplementation. In this study, the authors observed an increase in total cholesterol levels overall, LDL + VLDL cholesterol and phospholipids in the serum of the group that did not ingest *spirulina*. However, there was a significant reduction in the levels of these cholesterol fractions when the animals were supplemented with 16% *spirulina*.

Iwata *et al*. [17] observed that *spirulina *supplementation inhibited the increase of HDL - cholesterol, triglycerides, and phospholipids in the plasma. On the other hand, there was no statistical significance observed between the control group and the groups supplemented with *spirulina *when lipid levels in the liver were compared. Furthermore, the authors reported an increase in lipoprotein lipase enzyme activity in the animals that received *spirulina *supplementation.

The results found in the literature on the relationship between lipid profiles and *spirulina *intake need more controlled studies. Recently Cheong *et al*. [32] affirmed that the anti-hypercholesterolaemia mechanisms of *spirulina *are still not well understood, although some authors suggest that the addition of this alga into the diet diminishes the intestinal absorption of cholesterol as well as the re-absorption of bile acids in the ileum. Thus, they suggest that *spirulina *can be considered a functional food capable of reducing the levels of cholesterol and consequently preventing atherosclerosis.

Our results seem to corroborate the finding of Kato *et al*. [27], who show a reduction in the levels of serum cholesterol in animals supplemented with *spirulina*. However, the unexpected finding in our data was the reduction of HDL cholesterol found in the DSE group in relation to the DC group. The literature still contains conflicting findings in relation to this fraction of cholesterol and *spirulina *supplementation. Iwata *et al*. [17] suggest that *spirulina *can inhibit or augment HDL cholesterol, and it seems that this variable does not have a determined variable according to the modifications caused by the action of *spirulina*.

Corroborating the work of Blé-Castillo *et al*. [33], who observed that the administration of *spirulina *prevented an increase of total hepatic lipids (40%), our study found a positive effect from both exercise and a diet including *spirulina *when we verified the reduction in total lipids in hepatic tissue (in the DS, DSE and DE groups in comparison to the DC group) and the triglycerides of these same tissues (the DS group in comparison to the DC group).

According to the results found in the circulating lipid profile, it is possible to observe that the association between the effects of diet with *spirulina *and exercise seem to cause major changes. When analysed from this perspective, the HDL and LDL cholesterols were different for the DSE group in relation to the DC group. However, when we look at the specific effect of one of the conditions, the results point to the possibility that only *spirulina *causes modifications, causing a statistical difference in the LDL cholesterol. We understand that the positive effects found for hepatic and plasma levels are very important when we analyse lipid metabolism, and that *spirulina*, mainly when associated with exercise, seems to be an agent capable of causing interesting modifications in this metabolism.

By analysing the study of Moura *et al*. [34], we verified that the rats from the same breed used here (Wistar), non-diabetic and maintained with a balanced diet showed concentrations of total hepatic tissue lipids of 3.5 ± 0.4 mg% for sedentary animals and of 3.5 ± 0.5 mg% for water-exercised animals. Compared with these data, we report results that were approximately 117, 24, 21 and 18% higher in the DC, DS, DSE and DE groups, respectively. Thus, we can assert that hepatic steatosis was induced in the DC group and present evidence that the intervention of *spirulina *and/or exercise seems to reduce the accumulation of total hepatic lipids in diabetic rats, thereby attenuating hepatic steatosis.

## Conclusion

Based on our results, we conclude that *spirulina *intake can provide lower levels of circulating LDL cholesterol in comparison to aerobic training in diabetic Wistar rats. Furthermore, both *spirulina *intake and physical exercise cause an improvement in hepatic steatosis in these animals.

## List of abbreviations

DM-1: type 1 diabetes mellitus; DM-2: type 2 diabetes mellitus; NAFLD: non-alcoholic fatty liver disease; FFA: free fat acids.

## Conflicts of interest

The authors declare that they have no competing interests.

## Authors' contributions

All authors was responsible for the experimental design, data collection, statistical analysis and preparation of the manuscript. All authors worked read and approved the final manuscript.
